# *Taenia ovis*: an emerging threat to the Chinese sheep industry?

**DOI:** 10.1186/s13071-016-1700-5

**Published:** 2016-07-26

**Authors:** Yadong Zheng

**Affiliations:** 1 State Key Laboratory of Veterinary Etiological Biology, Key Laboratory of Veterinary Parasitology of Gansu Province, Lanzhou Veterinary Research Institute, CAAS, 1 Xujiaping, Yanchangbu, Lanzhou, 730046 Gansu China; 2 Jiangsu Co-innovation Center for Prevention and Control of Important Animal Infectious Diseases and Zoonoses, Yangzhou, 225009 China

**Keywords:** *Taenia ovis*, Sheep, Goat, Sheep industry

## Abstract

**Background:**

*Taenia ovis* is a tapeworm that is mainly transmitted between dogs and sheep. Although *T. ovis* infection is not a public health issue, it causes a great financial loss due to condemnation of carcasses.  The first outbreak of *T. ovis *infection in China occurred in 2015. Reassessment of adverse effects of *T. ovis* infection on Chinese sheep industry in future is necessary.

**Discussion:**

The first *T. ovis* outbreak in China suggests that the epidemic situation across the country is underestimated. For the transmission of *T. ovis*, many factors, including eggs, dogs and wild canids, human behaviours and sheep trade, should be seriously considered. In blocking the transmission chain, regular treatments of the infected dogs using anthelmintics play a crucial step, but at the moment it is difficult to be fully executed in China, largely due to the behaviours, customs and faith of local farmers. Moreover, combined with no clinical symptoms in the infected adult sheep and goats, the lack of pre-mortem diagnostic tools makes it harder to practice a national wide surveillance as well as inspection and quarantine in increasingly frequent free sheep trade activities in China, leading to an inability to restrict *T. ovis* infection into small areas. Furthermore, the ongoing campaigns against *Echinococcus granulosus* may have an adverse effect on control of *T. ovis* infection because of no consideration of a role of dogs in the transmission of the parasite.

**Conclusion:**

Lack of national epidemic data, pre-mortem diagnostic reagents and vaccines severely hampers the implementation of disease control campaigns and the restriction of *T. ovis* infection into small areas. Consequently, sheep and goats are at an increasing risk of *T. ovis* exposure and the possibility of large-scale outbreaks across China in future is possible, causing great adversity towards sheep industry.

## Background

*Taenia ovis* is a tapeworm that resides in the small intestine of dogs. With dog faeces, *T. ovis* eggs are released into the surrounding environment and develop into larvae predominately in body muscles, but occasionally in the liver, lung, kidney and brain, when sheep and goats ingest egg-contaminated water or forage. After nearly 1.5 months of development in the muscles, these larval parasites become infective [1], and dogs are infected with the ingestion of contaminated muscles or organs, thus completing a life cycle.

*Taenia ovis* is distributed across the world and its outbreaks have had a tendency to be sporadic in the past decade [2–5]. In 2015, a *T. ovis* outbreak was first reported in Gansu Province in the northwestern part of China, and more than one half of sheep in a farm were infected, with an economic loss of up to $16,000 [6]. Therefore, the reassessment of adverse effects of *T. ovis* infection on the Chinese sheep industry in future is necessary. Although *T. ovis* infection is not a public health issue, it hampers the development of the sheep industry by having an adverse effect on sheep trade and causing a great financial loss due to condemnations of carcasses [4, 7]. Considering multiple factors including the epidemiological features, lack of diagnostic tools and vaccines, a huge quantity of sheep and goats (> 146 million in 2007 in China) and traditional breeding management, sheep and goats are at a high risk of *T. ovis* exposure and it is potential to deteriorate the Chinese sheep industry in future due to the possibility of a wide spread of *T. ovis* infection.

## Discussion

Although the first outbreak does not rule out the possibility of imported cases, it may have resulted from the spread of *T. ovis* present in remote areas or regions in China [6], and also suggest that the epidemic situation across the country is underestimated. The assessment of the epidemiology of *T. ovis* is urgent because the lack of epidemic data makes it difficult and inefficient to implement disease control campaigns, such as an ongoing programme called Ovis Management Limited in New Zealand [7]. For the transmission of *T. ovis*, many factors, including eggs, dogs and wild canids, human behaviours and sheep trade, should be carefully considered. It was estimated that one adult worm could produce 70,000 ~ 250,000 eggs per day [7], posing a huge resource for pasture and water contamination. Although the developmental and maturation processes of eggs in the environment are largely unknown, they maintain infectivity for as long as one year on pasture [8]. The movement of sheep, wind, birds and rainwater may contribute to the dissemination of eggs from one place to another [7], which makes more flocks of sheep and goats at a risk of egg exposure. Therefore, effective control of the infected dogs, an original source of *T. ovis* eggs, plays a crucial step in blocking the transmission chain. The control measures include regular treatments of dogs with anthelmintics and a ban on feeding dogs with contaminated muscles or organs. It seems impossible to fully implement these measures in remote rural areas, especially in Gansu Province, Qinghai Province and Xinjiang Autonomous Region where people usually practice home-slaughter, and owners feed dogs with offal and are not willing to treat dogs with anthelmintics due to their customs and faith. These human behaviours are also considered to contribute, to a large degree, to the current high prevalence of cystic echinococcosis in China, caused by a small tapeworm, *Echinococcus granulosus*, that is also transmitted mainly between sheep and dogs [9]. Moreover, the role of wild canids in the spread of *T. ovis* remains elusive. Although *T. ovis* infection was reported in wolves, it is still controversial that wolves can act as definitive hosts [7, 10]. Coyotes have been suggested to transmit *T. ovis* [7], but it is still not yet verified. If wild canids, including wolves and foxes, are really involved in the transmission of *T. ovis* as definitive or reservoir hosts, the epidemic status becomes more complicated because of the full implementation of The Ecologic Restoration Plan in recent years in China, which favours returning farmland to forestry. Furthermore, increasing free animal trade activities in China would also accelerate the rapid spread of *T. ovis* from one place to another, making outbreaks unpredictable. Under these circumstances, the variables involved in transmission significantly increase the risk of exposure to *T. ovis* for both sheep and goats.

Except for *Taenia krabbei,* which is morphologically indistinguishable from *T. ovis* and is transmitted between carnivores and cervids [11], *T. ovis* is easily identified based on the morphological traits, host-preference and organotropism. Unfortunately, approaches for pre-mortem diagnosis of *T. ovis* infection in sheep are unavailable. At present, meat inspection is commonly used for identification of *T. ovis* infection. It should be noted that, as the sensitivity of meat inspection heavily relies on the intensity of infection, the true prevalence of *T. ovis* will be remarkably underestimated by utilization of abattoir data [7]. *Taenia ovis* infection in adult sheep and goats generally presents no clinical manifestations, which causes it to always be neglected. Combined with no clinical symptoms in the infected adults, the lack of pre-mortem diagnostic tools makes it harder to practice a national wide surveillance as well as inspection and quarantine in frequent free sheep trading in China. Therefore, the development of diagnostic reagents for pre-mortem detection of *T. ovis* infection, such as serological tests like enzyme-linked immunosorbent assay (ELISA), is urgently required.

One of the optimal control strategies is to vaccinate animals. However, although several antigens have been found to induce high protection against *T. ovis* infection even in field trials, no vaccines are commercially available up to now [12]. Treatment of infected sheep and goats using anthelmintics is likely unnecessary because *T. ovis* metacestode-contaminated carcasses are still subjected to condemnation rather than for human consumption. For the elimination of egg contamination, effective anthelmintics including praziquantel are available for the treatment of dogs, which is one of the core strategies of The Hydatid Control Plan (HCP) that is now being fully implemented in the echinococcosis-epidemic areas in China. It should be pointed out that the HCP’s implementation may have an adverse effect on eradication of *T. ovis* because owners are only educated not to feed dogs with offal including liver and lung, so they may feed dogs with cyst-containing carcasses. A lesson may be learnt from New Zealand, where the infectious rate of *T. ovis* was rather low prior to 1950 but increased substantially in the 1960’s, largely due to the implementation of anti-*E. granulosus* campaigns [7]. Therefore, it is highly recommended to make modifications of the on-going HCP strategies with full consideration of *T. ovis* transmission.

## Conclusion

Mainly due to lack of national epidemic data and pre-mortem diagnostic reagents, it is very difficult to efficiently implement disease control campaigns and restrict *T. ovis* infection to small areas. Consequently, the exposure of sheep and goats to *T. ovis* is notably increased and the possibility of large-scale outbreaks across China in future is possible, causing considerable problems for the sheep industry.

## Abbreviations

ELISA, enzyme-linked immunosorbent assay; HCP, Hydatid Control Plan
